# Influence of Anodizing Parameters on Tribological Properties and Wettability of Al_2_O_3_ Layers Produced on the EN AW-5251 Aluminum Alloy

**DOI:** 10.3390/ma15217732

**Published:** 2022-11-03

**Authors:** Mateusz Niedźwiedź, Marek Bara, Władysław Skoneczny, Sławomir Kaptacz, Grzegorz Dercz

**Affiliations:** Institute of Materials Engineering, Faculty of Science and Technology, University of Silesia in Katowice, 41-500 Chorzów, Poland

**Keywords:** aluminum alloys, oxide layer, contact angles, surface free energy, tribology

## Abstract

The article presents the effect of anodizing parameters of the EN AW-5251 aluminum alloy on the thickness and roughness of Al_2_O_3_ layers as well as their wettability and tribological properties in a sliding combination with the T7W material. The input variables were the current density of 1, 2, 3 A/dm^2^ and the electrolyte temperature of 283, 293, 303 K. The tribological tests were performed on the T-17 tester in reciprocating motion, in conditions of technically dry friction. The tests were carried out on a 15 km road with a constant average slip speed of 0.2 m/s and a constant unit pressure of 1 MPa. The measurement of the wettability of the layers was performed using the sitting drop method, determining the contact angles on the basis of which the surface free energy was calculated. The profilographometric measurements were made. The analysis of the test results showed that the anodizing parameters significantly affect the thickness of the Al_2_O_3_ layers. The performed correlation analysis also showed a significant relationship between the roughness parameters and the wettability of the surface of the layers, which affects the ability to create and maintain a sliding film, which in turn translates into sliding resistance and wear of the T7W material. The analysis of friction and wear tests showed that the layer with hydrophobic properties produced at a current density of 1 A/dm^2^ in an electrolyte at a temperature of 283 K is the most favorable for sliding associations with T7W material.

## 1. Introduction

Aluminum, after iron, is one of the most widely used metals in industry and the most abundant metal in the Earth’s crust. Its use is increasing, which makes it the metal of the future [1,2]. Pure aluminum is a material with low strength properties (including low hardness); therefore, in order to improve its final properties, chemical elements are added, depending on the purpose of application, to form aluminum alloys [3]. The production of aluminum alloys is carried out mainly by adding such elements as: iron, copper, magnesium, silicon, zinc, or manganese. Additions of alloying elements affect the strength of aluminum—especially in combination with cold or hot deformation hardening. Aluminum alloys with a high magnesium content and small admixtures of other elements (5xxx series) are very well suited for anodizing. They are characterized by high plasticity and corrosion resistance [4,5,6,7].

The oxide layers produced using the anodic oxidation (anodization) method allowed to improve both the physical and surface properties of aluminum alloys (hardness, corrosion, and abrasion resistance) [8]. The improvement of surface properties of aluminum alloys has resulted in their much wider application in various industries, such as machinery, aviation, automotive, electronics, and even in nanotechnology (production of nanowires and membranes for sensors) [9,10,11]. The oxide layers used in kinematic sliding nodes are suitable for cooperation with metals and ceramics (limited lubrication) and with plastics (technically dry friction). A very important element determining the tribological cooperation in the case of technically dry friction is the formation of a lubricating layer between the cooperating elements. It contributes to a significant reduction of friction, which results in lower wear of cooperating elements [12]. During technically dry friction, the cooperation of Al_2_O_3_ layers is carried out with plastics showing film-forming properties, which include graphite, PTFE, and MoS_2_. Currently, the materials containing PTFE (polytetrafluoroethylene), which are characterized by a very low coefficient of friction, have the greatest application in the sliding elements of industrial devices and machines. Examples include T5W and T7W [13].

Currently, great importance is attached to the energy state of materials. The hydrophobic properties of materials (repelling water molecules), and therefore with low surface wettability, allow them to be used very often in surface engineering, most often as anti-icing or self-cleaning materials [14,15]. The opposite of hydrophobic materials are hydrophilic materials (attracting water molecules) with high surface wettability. They can be used in biomaterials and photovoltaic panels [16,17,18]. The energy state of the surface also has a significant influence on the mechanical and tribological properties. The contact angle of the material, and thus the surface free energy, significantly contributes to the improvement of material lubrication and reduction of friction [19,20,21]. More and more attention has been paid in recent times to the tribology of surface layers in relation to the energy state of the surface. This is due to the influence of surface free energy on the course of tribological processes and on such phenomena as abrasive wear and the formation of a sliding film [22]. A hydrophobic surface is a surface with a contact angle greater than 90°, measured with water. A surface with a contact angle for water greater than 150° is conventionally called a super-hydrophobic surface. When the contact angle of the surface with water is less than 90°, we speak of hydrophilicity, i.e., the opposite of hydrophobicity [23]. The analysis of world literature shows that the vast majority of scientific research on the wettability and energy state of Al_2_O_3_ layers mainly concerns the studies of Al_2_O_3_ layers after modification. No publications regarding the influence of anodizing parameters have been noticed (electrolyte temperature, current density) on the wettability, and hence the energy state of the oxide layers, which was the aim of the presented research. Then, the impact of the layers’ wettability on their tribological parameters (friction coefficient, mass loss) was investigated.

## 2. Materials and Methods

### 2.1. Research Material

As a substrate for the production of Al_2_O_3_ layers, samples made of aluminum sheet of the EN AW-5251 alloy were used. The process of preparing samples for anodizing consisted of leveling the edges of the samples and removing any unevenness that appeared when cutting the material with a stream of water from the rolled sheet. The samples were sequentially cleaned of any contamination caused by processing using an ultrasonic washer. Distilled water was used as the bath liquid. The next step was to glue the samples with a two-component epoxy adhesive that does not react with the acid of the electrolyte and the chemicals for etching and neutralization. Sample sticking is used to reduce the anodizing surface only to the necessary (needed for testing). The last step before the actual process was to subject the samples to etching, immediately before anodizing, in a 5% KOH solution for 20 min and neutralization in a 10% HNO_3_ solution for 5 min. The processes were carried out at room temperature.

**Figure 1:**
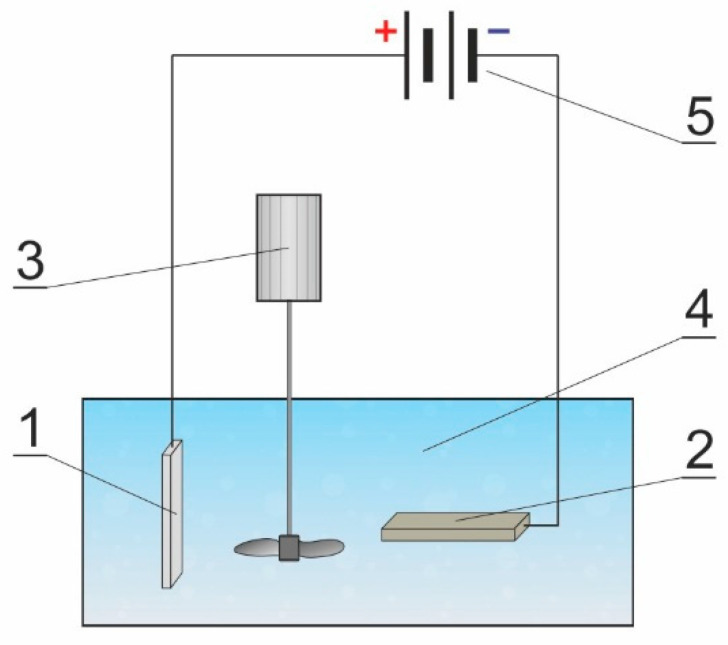
Diagram of the anodizing system used during the production of the oxide layer: 1—aluminum alloy EN AW-5251 (anode), 2—lead plate (cathode), 3—mechanical stirrer, 4—electrolyte, 5—power supply.

In order to produce Al_2_O_3_ layers, samples from the EN AW-5251 aluminum alloy were electrolytically anodized with the direct current method using a stabilized GPR-25H30D power supply. The surface of the aluminum alloy on which the layer was produced was the anode, the cathode was a lead plate with dimensions corresponding to the dimensions of the aluminum samples. In anodized oxidation processes, electrolyte-resistant metals (usually stainless steel or lead) are used as the cathode. As a result of many years of experience of our research team, we use lead sheet for the production of Al_2_O_3_ in order to compare the test results with previous tests. The diagram of the anodizing system used is shown in Figure 1.

The anodizing process was carried out in a three-component electrolyte consisting of an aqueous solution of 18% H_2_SO_4_ (33 mL/L), C_2_H_2_O_4_ (30 g/L), and C_8_H_6_O_4_ (76 g/L). During the anodizing process, the solution was mixed with a mechanical stirrer at a speed of 100 rpm, changing the direction of rotation after every 10 min. The anodizing parameters were selected on the basis of the experimental plan (Table 1).

Table 1 presents the total experimental plan for two input variables with three variable values. Based on the matrix of the total experiment design, the parameters for anodizing the aluminum alloy were selected. The input variables were current density of 1, 2, 3 A/dm^2^ and electrolyte temperature of 283, 293, 303 K. The anodizing time for all samples was 20 min. After the completion of the anodizing process, the samples were rinsed in distilled water for 60 min.

The T7W material was used as a tribo partner in tribological tests. The T7W material is a composite made on the basis of PTFE with the dispersion phase in the form of powdered technical carbon. The material is widely used in hydraulic and pneumatic piston-cylinder systems, as a material for sealing rings used, for example, in pneumatic cylinders.

### 2.2. Research Methodology

Microscopic examinations were carried out using a Hitachi S-4700 scanning electron microscope (Hitachi, Tokyo, Japan). Surface morphology images were taken at 50,000× magnification to observe the nanopores. The anodic oxide layers are poorly conductive, so they charge electrically during operation of the electron beam, which contributes to incorrect observation. For proper observation, the layers were sprayed with carbon using a turbomolecular carbon sputtering machine. The carbon layer enables the bouncing electrons to be discharged and carried away during the research.

Chemical composition tests were carried out for both cross-sections. The chemical composition of the layers was investigated using the NoranVantage EDS (Energy Dispersive X-ray Spectroscopy) system connected to the Hitachi S-4700 scanning microscope. The EDS tests for cross-sections were carried out on metallographic specimens.

In order to obtain the phase composition, diffraction tests (XRD) were carried out on the X-Pert Philips diffractometer. The device operated at the parameters of 30 mA and 40 kV (PANalytical, Almelo, The Netherlands). During the research, a vertical goniometer, Euler cradle, and a copper X-ray source (λCuKα) were used with a wavelength of 1.54178 Å. X-ray diffraction patterns were recorded (GIXD) from an angle of 2θ 10–100° in steps of 0.05° for angles of incidence α = 0.20°; 0.30°; 0.50°; 1.00°; 1.50°; 2.50°; 5.0°. Finally, the angle α = 0.20° was chosen to illustrate.

The thickness of the oxide layers was measured by the contact method using a Fischer Dualscope MP40 instrument (Helmut Fischer GmbH + Co. KG, Shin-delfingen, Germany). The device measures using the eddy current method. On the surface of each sample, 10 measurements were made, which were then used to calculate the average value of the thickness of the oxide layer with deviations.

**Figure 2:**
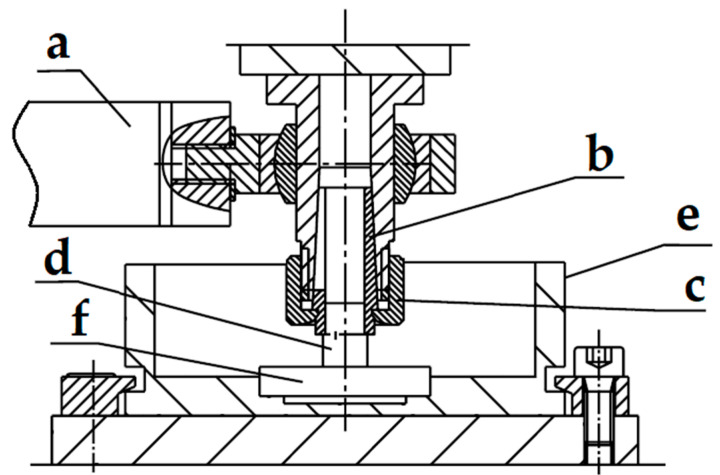
Diagram of the friction junction of the T-17 tester: (a) Force sensor, (b) collet, (c) clamping nut, (d) upper pin, (e) reservoir, (f) plate.

The tribological tests were carried out for the friction pair of the pin-plate type on the T-17 tester (Figure 2) in a reciprocating motion [24]. A constant slip speed of 0.2 m/s and a constant unit pressure of 1 MPa were used. The tests were carried out in dry technical friction conditions, at a constant ambient temperature of 298 ± 1 K and a relative air humidity of 40 ± 10%. The friction path during the tribological test was 15 km. The friction force was measured with the Spider 8 transducer, which allows the data to be exported to a computer and saved using Catman 4.5 software. The friction coefficients were calculated from the stabilized range of friction force values. The mass loss of the pin was determined by the weight method.

The profilographometric measurements were carried out in order to determine the roughness parameters before and after the tribological test. Measurements were made using a Form TalySurf Series 2.50i contact profilegraphometer (Taylor Hobson Ltd., Leicester, UK).

The wettability of the layers was measured using the sitting drop method at a constant ambient temperature of 298 K. Four liquids were used, two polar (water and glycerine) and two non-polar (α-bromonaphthalene and diiodomethane). On each of the Al_2_O_3_ layers using a 0.5 µL micropipette, 10 drops of each liquid were applied along the entire length of the sample. After each drop was applied, it was photographed with a camera and exported to a computer. The software used enables the automatic measurement of the drop’s contact angle by marking the three extreme points of the drop in the picture. The smallest and largest values of the angles were rejected, the remaining eight were used to calculate the average value of the contact angle for a given surface. The surface free energy was calculated using the Owens–Wendt method using the contact angles for polar (distilled water) and non-polar (α-bromonaphthalene) liquids using the following Equation (1):

γ_s_ = γ_s_^d^ + γ_s_^p^, (1)

where γ_s_ responsible for the free surface energy of a solid, γ_s_^d^ is the component of surface free energy dispersion, γ_s_^p^ is the component of surface free energy dispersion of materials.

## 3. Results and Discussion

### 3.1. Structure of the Oxide Layer

DC anodizing begins with a rapid increase in voltage, which is necessary to break through the natural barrier layer, after reaching the maximum value (critical voltage) it begins to decrease [25]. Reaching the minimum value results in the reconstruction of the barrier layer at the oxide-electrolyte interface. In the next stage of the process, pores are formed and deepened, leading to the growth of the oxide layer. This is caused by an increase in tension over time, but much slower than at the beginning of the process. The value of the critical voltage decreases with increasing electrolyte temperature, and it increases with increasing current density [26,27]. The formation of the oxide layer during the DC anodizing process proceeds according to the following processes: barrier layer formation, propagation of disturbances in the oxide layer (microcracks), pore formation process, growth of the oxide layer along with the deepening of the pores.

**Figure 3:**
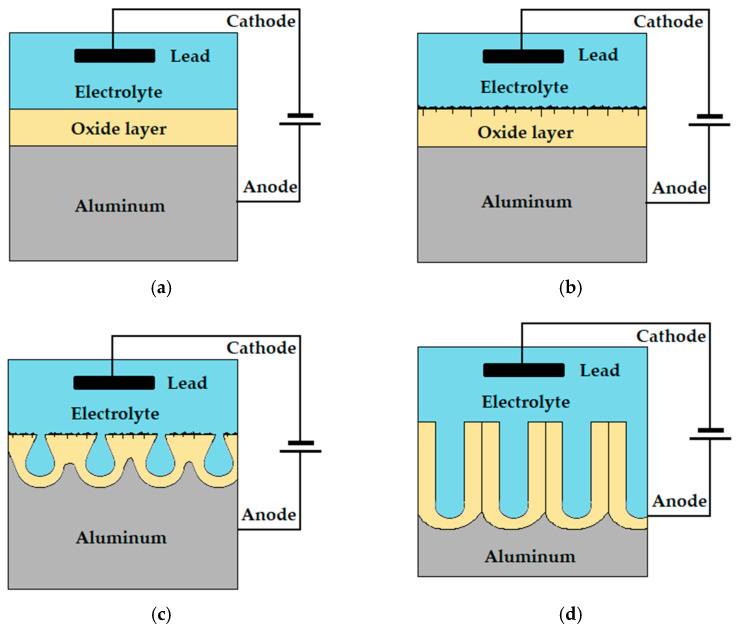
The steps in the formation of an oxide layer during DC anodizing: (**a**) barrier layer formation, (**b**) propagation of disturbances in the oxide layer (microcracks), (**c**) pore formation process, (**d**) growth of the oxide layer along with the deepening of the pores.

The graphical interpretation of the processes during DC anodizing, depending on the voltage changes is shown in Figure 3 [27,28].

**Figure 4:**
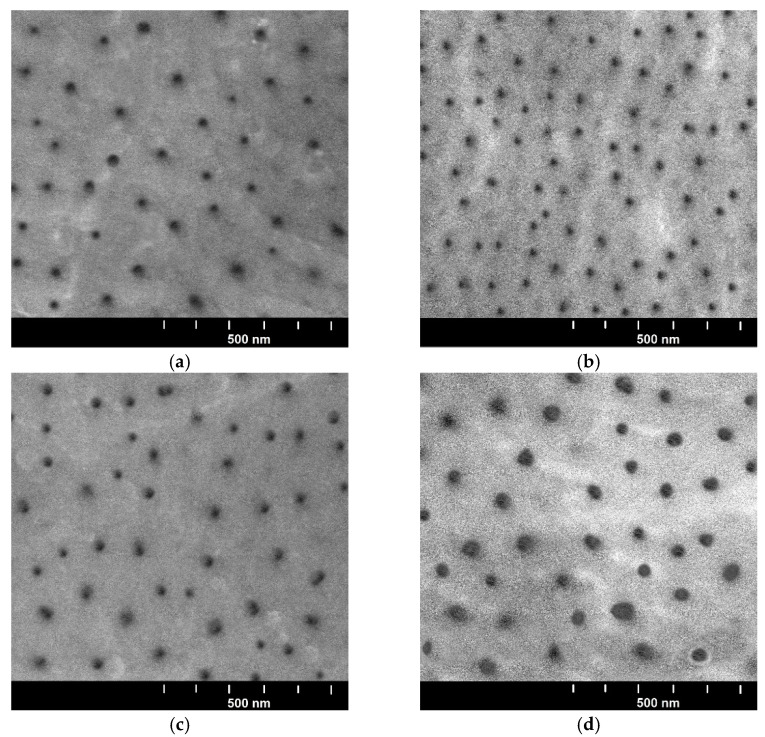
Images of surface morphology of oxide layer: (**a**) sample A, (**b**) sample C, (**c**) sample E, (**d**) sample G.

Figure 4 shows the surface images of the oxide layer for four samples with different properties. In order to observe nanopores characteristic of the oxide layers, a magnification of 50,000× was used.

The porosity of the oxide layers is a characteristic feature of the oxide layers resulting from the manufacturing parameters [29].

**Figure 5:**
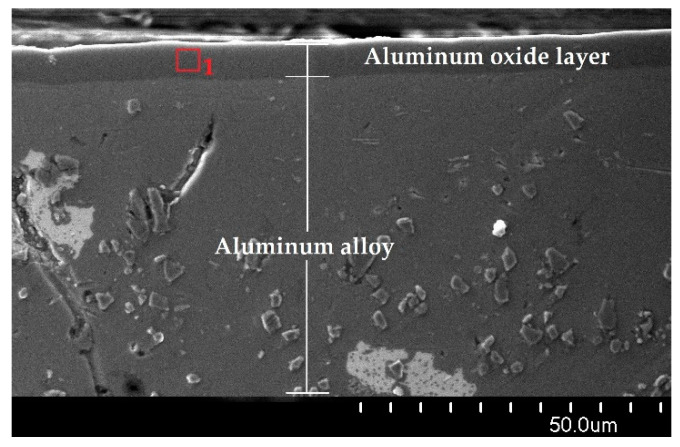
Cross-section of the oxide layer at a magnification of 1000× of sample A.

Figure 5 shows a cross-section of the oxide layer of sample A at 1000× magnification. In addition, the photo shows the oxide layer and EDS analysis site for sample A.

The chemical composition tests carried out with an EDS spectrometer on the cross-sections of the layers showed the aluminum content at the level of 55.9% and the oxygen content at the level of 44.1% for all tested layers. The chemical composition of the oxide layers should be 52.9% Al and 47.1% O. However, only the EDS analysis carried out in the very center of the layer will show a chemical composition similar to the stoichiometric calculation. The decrease in the aluminum content and the increase in the oxygen content along the thickness of the Al_2_O_3_ layer is due to the change in the stoichiometry of the resulting layer.

**Figure 6:**
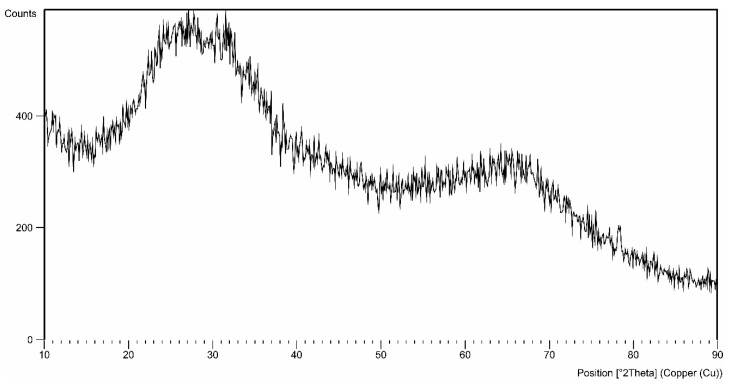
GIXD pattern for the angle of incidence α = 0.20 obtained.

Figure 6 shows the GIXD pattern for sample A produced at 1 A/dm^2^, at 283 K.

Aluminum oxide exists in an amorphous form and in several crystalline forms. Only the α-Al_2_O_3_ form is a stable crystalline form. GIXD tests carried out for layers produced by the electrochemical method in the ternary electrolyte showed that the obtained coating is amorphous, regardless of the production conditions and can be considered as an amorphous X-ray pattern. The phase analysis of the examined layers showed in all cases the presence in the angular range of 20–45 degrees 2θ of the amorphous “halo”, which is characteristic for the oxide layers produced by the electrochemical method (Figure 5). In no case were there any reflections belonging to the elements with a crystalline structure.

### 3.2. The Thickness of the Oxide Layer

Table 2 shows the average measurement results of the thickness of the oxide layers produced in accordance with the total plan with standard deviations.

The anodizing parameters affect the thickness of the oxide layer. The highest thickness of the Al_2_O_3_ layer was measured for sample B produced at a current density of 3 A/dm^2^ and an electrolyte temperature of 283 K, and the lowest for sample C produced at a current density of 1 A/dm^2^ and an electrolyte temperature of 303 K.

**Figure 7:**
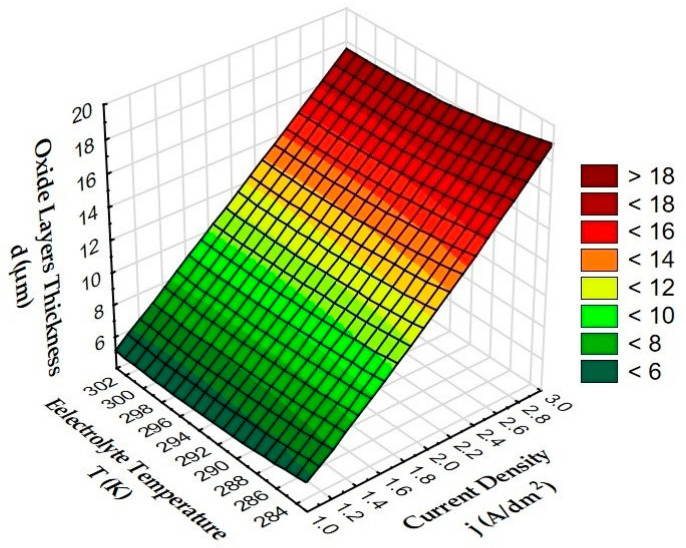
Dependence of the oxide layers thickness of oxide layers on the current density and electrolyte temperature.

Figure 7 shows the effect of anodizing parameters (current density and electrolyte temperature) on oxide layer thickness.

Taking into account the thickness measurements carried out, it can be concluded that the anodizing parameters (current density and electrolyte temperature) in the constant process time have a significant impact on the thickness of the Al_2_O_3_ layers. As the current density increased at a constant electrolyte temperature, a significant increase in the thickness of the layer was observed. The increase in the thickness of the oxide layer is caused by the increasing value of the electric charge. The increase in the temperature of the electrolyte, at a constant current density, reduces the thickness of the layer, which can be explained by the increasing secondary solubility of the Al_2_O_3_ layer by the electrolyte with a higher temperature.

### 3.3. Tribological Tests

**Figure 8:**
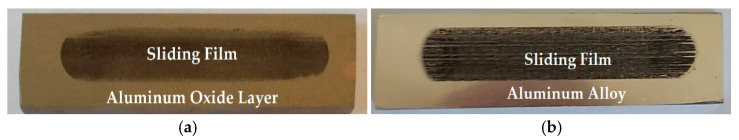
The surfaces of samples with a sliding foil applied as a result of tribological tests: (**a**) Sample B, (**b**) sample Al.

As a result of tribological cooperation, a sliding film made of plastic was obtained on the surfaces of the tested samples. Examples of samples with the applied film are shown in the Figure 8.

Table 3 shows the values of the friction coefficient µ during the sliding cooperation of Al_2_O_3_ layers and the surface of the EN AW-5251 aluminum alloy with the T7W mandrel.

The anodizing parameters influence the values of the friction coefficient µ. The highest friction coefficient among Al_2_O_3_ layers was determined for sample C produced at a current density of 1 A/dm^2^ and an electrolyte temperature of 303 K, while the lowest for sample G produced at a current density of 2 A/dm^2^ and an electrolyte temperature of 283 K. The highest coefficient of friction µ, among the tested surfaces, was determined for the EN AW-5251 aluminum alloy without anodizing modification. Therefore, it can be concluded that the anodizing process contributes to the improvement of the tribological cooperation in the reciprocating motion between the aluminum alloy (EN AW-5251) and the T7W material.

**Figure 9:**
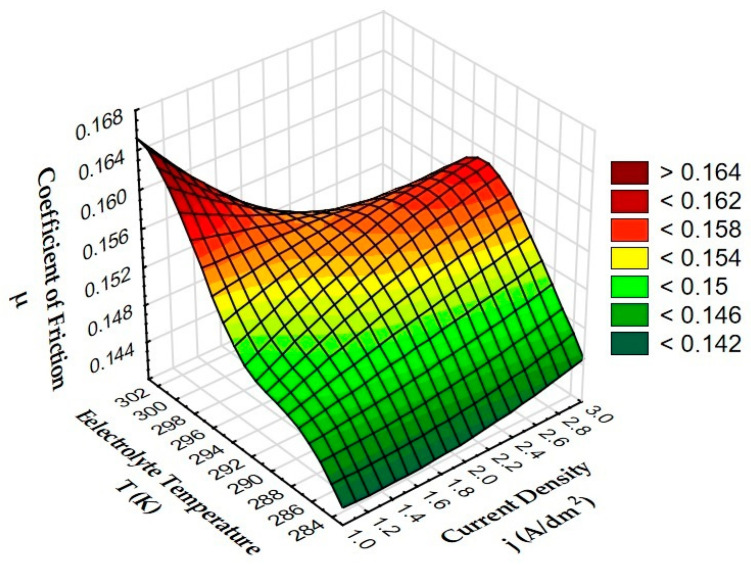
Dependence of the friction coefficient µ of oxide layers on the current density and electrolyte temperature.

Figure 9 shows the effect of anodizing parameters (current density and electrolyte temperature) on the friction coefficient µ of the Al_2_O_3_ layer cooperation with T7W material.

Based on the graph of the dependence of the friction coefficient µ on the production parameters (current density and electrolyte temperature), it was found that high values of the friction coefficient occur for the layers produced at an electrolyte temperature of 293 K, increasing gradually with increasing current density. The lowest values were observed for the lowest electrolyte temperature of 283 K, where the values of the friction coefficient also increase with increasing current density. The highest value of the coefficient of friction µ with the T7W material was observed for the sample produced at a current density of 1 A/dm^2^ and an electrolyte temperature of 303 K (characteristic elevation in the diagram). The increase in the current density during anodizing at an electrolyte temperature of 303 K causes a gradual decrease in the coefficient of friction. Summing up, it can be stated that the best sliding cooperation (due to frictional forces) of T7W material occurs with Al_2_O_3_ layers produced at the lowest electrolyte temperature (283 K) gradually from the current density of 1 A/dm^2^ (the lowest value). Table 4 shows the mass loss of T7W material during sliding cooperation with the Al_2_O_3_ layer and the EN AW-5251 aluminum alloy.

The anodizing parameters also significantly affect the mass loss of the material during the tribological run. The highest mass loss was measured for sample E produced at a current density of 1 A/dm^2^ in an electrolyte with a temperature of 293 K. The lowest mass loss was determined for sample H anodized at a current density of 2 A/dm^2^ in an electrolyte at a temperature of 303 K. During tribological cooperation of the alloy aluminum EN AW-5251 with T7W material, there is a significant wear of the material mandrel (0.6 mg)—almost four times higher than for the H sample with the Al_2_O_3_ layer with the lowest wear (0.16 mg).

**Figure 10:**
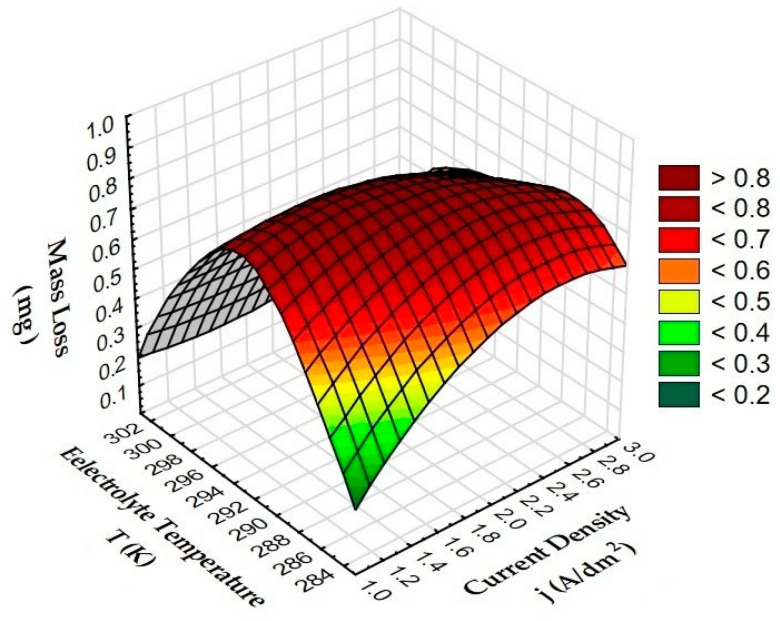
Dependence of mass loss of T7W material on the current density and electrolyte temperature during anodizing.

Figure 10 shows the effect of anodizing parameters (current density and electrolyte temperature) on the mass loss of the T7W pin.

The analysis of the T7W pin mass loss relationship presented in three-dimensional diagrams shows that high wear values occur for layers produced in the electrolyte at a temperature of 293 K (>0.5 mg). The lowest wear of T7W material (<0.4 mg) was measured for Al_2_O_3_ layers produced in the electrolyte at a temperature of 303 K (the lowest value among all samples for 2 A/dm^2^, 0.16 mg). Low value of mass loss (<0.2 mg) was also observed for sample A produced in 1 A/dm^2^ at 283 K (characteristic decrease in the diagram). Taking into account the friction and wear tests, it can therefore be concluded that these are the best parameters for the production of Al_2_O_3_ layers for sliding associations with the T7W material.

### 3.4. Surface Roughness Tests

Table 5 presents selected amplitude parameters (Rq—mean amplitude profile deviation from the mean line along the measuring or elemental section, Rsk—profile asymmetry coefficient, Rku—flattening coefficient) having the greatest impact on tribological cooperation and parameters of the load curve (Abotta Fireston) Rk—reduced roughness height, Rpk—reduced roughness profile elevation, Rvk—reduced roughness profile cavity height before and after tribological tests.

By analyzing both the amplitude parameters and the load capacity curve (Abott Fireston) before the tribological test, one can notice large differences resulting from the parameters of anodizing of the aluminum alloy. There is a significant relationship between the increase in electrolyte temperature and the increase in the parameters of the load-bearing curve Rku and Rpk. The Pearson correlation coefficient between the electrolyte temperature and these parameters is above 0.82, so we can speak of a high co-relation and significant dependence. The extreme amplitude parameters (the highest Rq, Rku, and the lowest Rsk) were observed for sample H, which was characterized by the lowest material wear during the tribological test. This sample is also characterized by high parameters of the load capacity curve, in particular the Rvk parameter, which is responsible for keeping the sliding film on the friction surface. Sample E, which is characterized by the highest material wear among the oxide layers, has high amplitude parameters Rsk and Rku and low parameters of the load capacity curve (Rk and Rvk). Such a difference in the dependence of the above parameters resulted in a different nature of the overlap of the sliding film on the surfaces of samples H and E, which in turn translated into differences in the wear of T7W material during tribological tests with these samples. The aluminum alloy sample showed a low roughness surface, it was characterized by very low amplitude parameters Rq and Rku, high Rsk parameter, and the lowest values of the load-bearing capacity curve parameters (Abott Fireston) among the tested samples, even several times lower compared to the oxide layers, which resulted in difficulties in creating a sliding film. 

The parameters after the tribological test underwent significant changes. Taking into account the amplitude parameters, the Rq values for all Al_2_O_3_ layers decreased, in the case of aluminum alloy it increased almost 10 times. The profile asymmetry coefficient (Rsk) increased only for three samples (A, F, I), for the others there was a decrease (over 3 times for aluminum alloy). The Rku parameter after the tribological test increased for samples: A, B, C and G, for all other samples there was a decrease in this parameter. While analyzing the parameters of the load curve, very interesting conclusions were noticed: the Rk parameter decreased significantly for all oxide layers, but for the aluminum alloy, it increased more than 10 times. The situation is similar for the Rpk parameter, its significant decrease was also observed for almost all layers (except for sample A) where the increase was insignificant and over 10-fold increase for the aluminum alloy. The reduced height of the roughness profile cavity (Rvk) after the tribological test decreased for almost all oxide layers (it increased slightly for sample A), and also increased over 15 times for the aluminum alloy. Sample H, which showed the lowest material wear during the tribological test, was characterized by a high Rq parameter, the highest Rku and one of the lowest Rsk parameters among the tested. The layer also has high parameters of the load capacity curve (Rk, Rpk, and Rvk) influencing the sliding cooperation and referring to the bearing nature of the layer. The parameters are completely different for the oxide layer marked with the symbol E, which is characterized by high material wear during friction. It has very low amplitude parameters (Rq and Rku) and high Rsk. Taking into account the parameters of the load curve, the sample has the lowest parameters Rk and Rpk and one of the lowest Rvk. On the basis of these values, it can be concluded that the conducted friction tests contributed to the formation of a sliding film on the sample surface, which will translate into very good tribological cooperation of the above layer, high abrasion resistance, and high load capacity of the layer.

**Figure 11:**
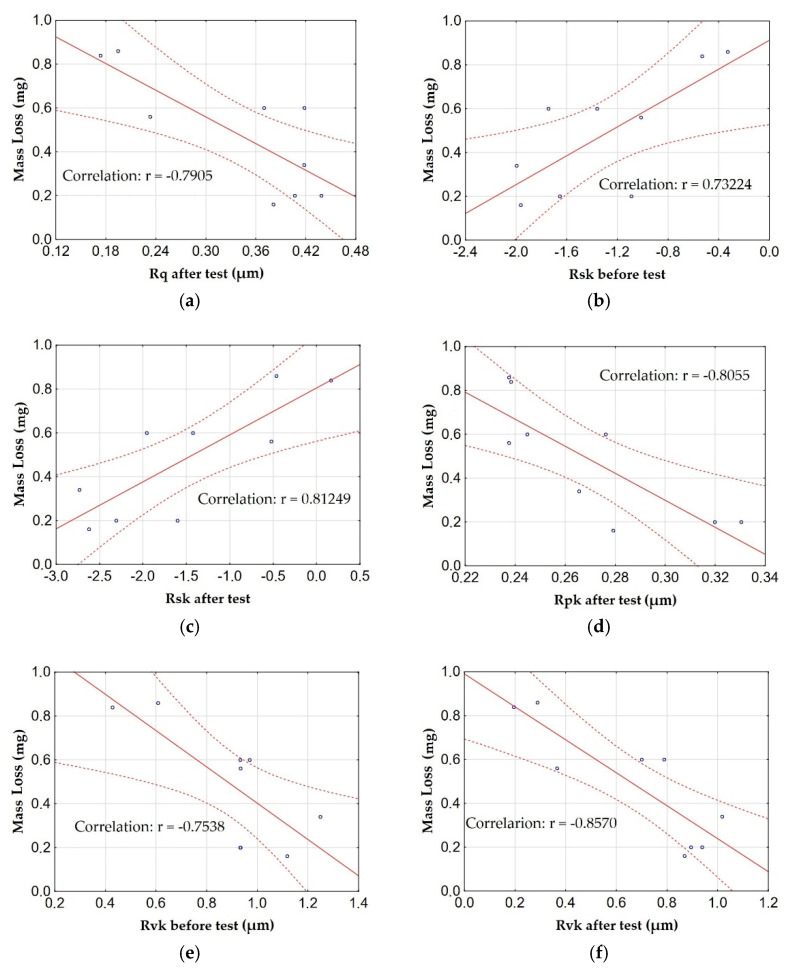
Relationships between the mass loss of T7W material and: (**a**) the Rq parameter after the test, (**b**) the Rsk parameter before the test, (**c**) the Rsk parameter after the test, (**d**) the Rpk parameter after the test, (**e**) the Rvk parameter before the test, (**f**) with the Rvk parameter after the test.

Figure 11 shows the graphs of the relationship (correlation) between the mass loss of T7W material during the tribological test and the amplitude parameters and the load capacity curve with the highest correlations. The matching characteristics (middle line) and the regression stripes (side lines) defining the confidence intervals are marked in the figures showing the relationships.

Taking into account the correlation diagrams between the mass loss of the material during the tribological test and the amplitude parameters and the load capacity curve, a very high correlation (very large relationship) between the mass loss and the parameters Rsk, Rpk and Rvk (after friction) can be noticed. A high correlation (significant dependence) between the mass loss and the parameters Rsk and Rvk (before friction) as well as Rq (after friction), Rsk (before) Rvk (before) was also noticed. In the case of the Rsk parameters before and after friction, it can be stated that the increase in mass loss occurs with an increase in the above-mentioned parameters. Taking into account the parameter Rvk before friction and the parameters Rq, Rpk, Rvk after friction, it can be stated inverse proportionality to the mass loss, in other words, an increase in parameters results in a decrease in material wear. This indicates a strong relationship between the surface roughness and the wear of the material during friction. 

**Figure 12:**
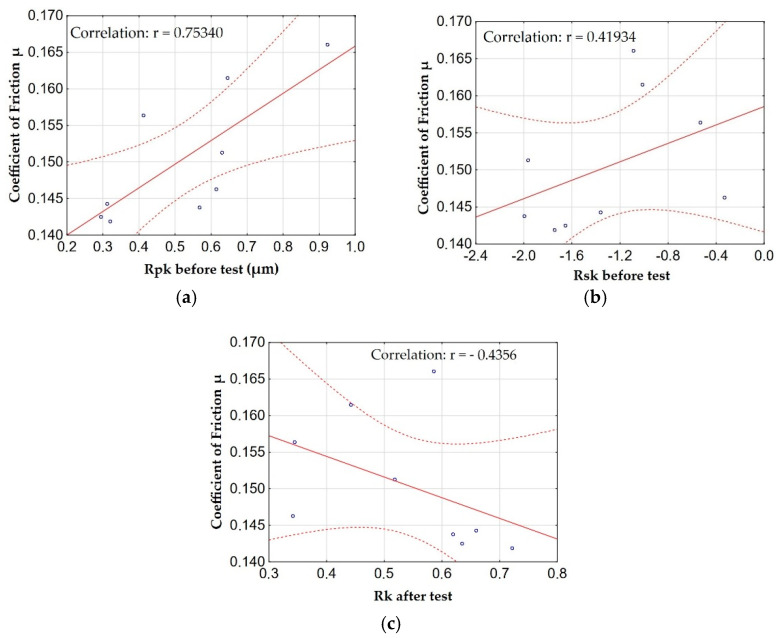
Relationships between the coefficient of friction μ and: (**a**) the Rpk parameter before the test, (**b**) the Rsk parameter before the test, (**c**) the Rk parameter after the test.

Figure 12 shows the graphs of the relationship (correlation) between the friction coefficient µ and the amplitude parameters and the load-bearing curve with the highest correlations.

**Figure 13:**
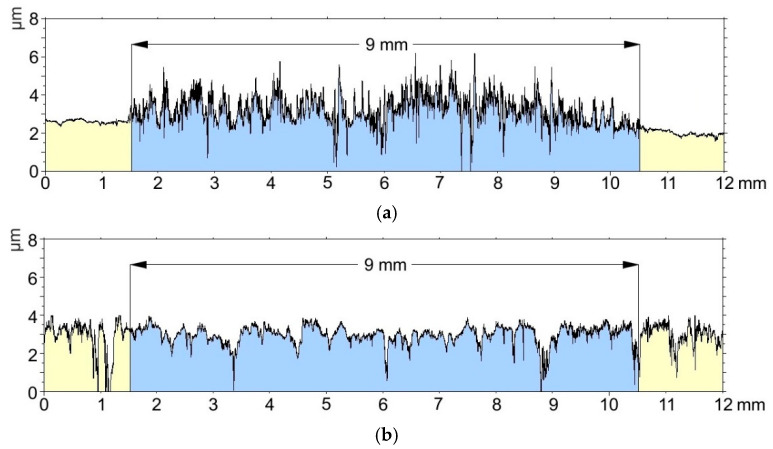
Roughness profiles: (**a**) aluminum alloy EN AW-5251, (**b**) Al_2_O_3_ layer.

The analysis of the correlation of the friction coefficient µ showed that the Rpk parameter before the tribological test shows a high correlation (significant relationship) with the friction coefficient µ. On the other hand, the parameters Rsk before the tribological test and the parameter Rk after the tribological test show a moderate correlation (significant relationship) with the friction coefficient µ. The Rpk and Rsk parameters before the tribological test are characterized by a positive correlation, in other words, the change in the friction coefficient µ is proportional to the change in parameters. The values of the Rk parameter after the tribological test are inversely proportional to the friction coefficient µ. Figure 13 shows the roughness profile for the aluminum alloy EN AW-5251 and the Al_2_O_3_ layer (sample F).

Based on the analysis of the roughness profiles for the aluminum alloy EN AW-5251 without modification and the Al_2_O_3_ layer, significant differences are noticeable. Tribopartner (T7W material) with a diameter of ϕ9 mm coincides with the width of the sliding film marked in Figure 6. Before the tribological test, aluminum alloy is characterized by low roughness, but cooperation with the material contributed to a significant increase in roughness and the appearance of a significant number of peaks on the surface, which results in a higher coefficient of friction and uneven overlapping of the sliding film on the friction surface. The sample with the oxide layer before friction had a much greater roughness than the aluminum alloy, but the tribological test influenced the smoothing of the sample and filling the cavities in the profile through an evenly superimposed sliding film.

**Figure 14:**
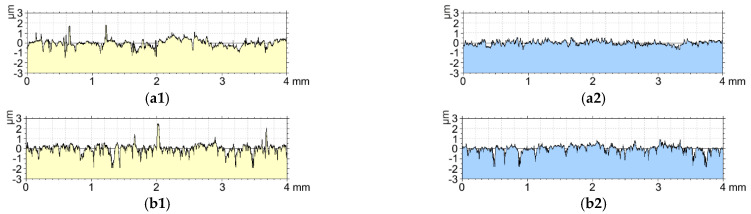
Roughness profiles: (**a**) sample E, (**b**) sample H, **1**—before the tribological test, **2**—after the tribological test.

Figure 14 shows the roughness profiles before and after the tribological test for the oxide layers with the highest material mass loss (sample E) and the lowest material mass loss (sample H).

By analyzing the roughness profiles for the oxide layers with the highest (sample E) and the lowest material wear (sample H), interesting relationships can be observed. At first glance, you can see a much greater roughness for sample H, it has a much greater number of recesses in the profile. The tribological test contributed to the smoothing of the vertices for both the first and the second layer. However, sample H, whose material wear as a result of the tribological test was over 5 times lower than that of sample E, is still characterized by a significant number of cavities in the profile, which proves that they are not completely filled with the sliding film. The lower wear can be explained by a much better retention of the sliding film in the cavities, thanks to which a self-lubricated layer is formed. A much larger number of cavities after the tribological test for sample H may indicate a much longer time to fill them with a sliding film. In turn, the higher coefficient of friction can be attributed to the higher surface roughness.

**Figure 15:**
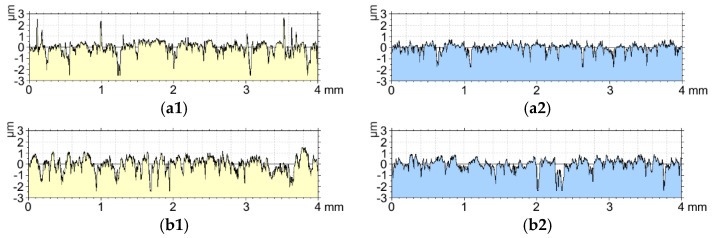
Roughness profiles: (**a**) sample C, (**b**) sample G, **1**—before the tribological test, **2**—after the tribological test.

Figure 15 shows the roughness profiles before and after the tribological test for the oxide layers with the highest coefficient of friction μ among the oxide layers (sample C) and with the lowest coefficient of friction (sample G).

When analyzing the roughness profiles of Al_2_O_3_ layers with the highest (sample C) and the lowest (sample G) friction coefficient µ, a significant influence of the roughness on this value was observed. The roughness profile of sample C, which was characterized by the highest coefficient of friction µ, has significantly higher peaks and a greater number of peaks than sample G. As a result of the tribological test, these peaks were smoothed, but their abrasion increased the friction coefficient. The profile of sample C after friction also has a smaller number of cavities, most probably as a result of sealing them with T7W material with the products of tip wear, which negatively influenced the friction process. In the case of sample G, larger cavities after friction are visible, which has a positive effect on maintaining the sliding film and reducing the coefficient of friction µ.

### 3.5. Measurements of Wettability and Calculation of Surface Free Energy

**Figure 16:**
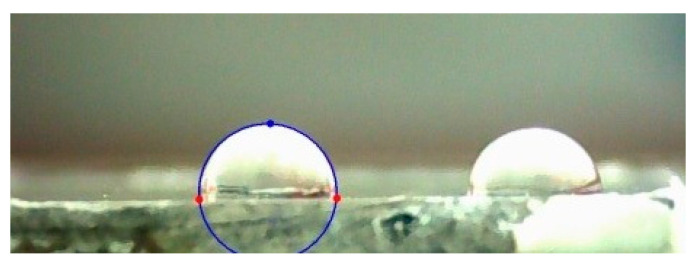
Sample photo of a drop on the surface of the G sample layer.

Table 6 shows the contact angle values measured with distilled water and α-bromonaphthalene. Table 7 lists the contact angle measurements with glycerin and diiodomethane. Examples of drops on the surface of layer G (with the greatest contact angle) are shown in Figure 16.

**Figure 17:**
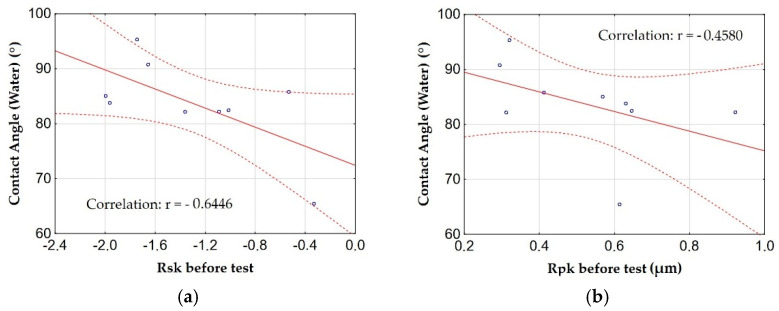
The relationship between the contact angle measured by water and: (**a**) the Rsk parameter, (**b**) the Rpk parameter.

Figure 17 shows graphs of the relationship (correlation) between the contact angle measured by water and the amplitude parameters and the load-bearing curve with the highest correlations.

By analyzing the correlation between the contact angles measured by water, the amplitude parameters and the load-bearing curve, it can be concluded that the roughness of the surface has a significant impact on its wettability. There is a high correlation (significant relationship) between the Rsk parameter and the contact angle for water, and a moderate correlation (significant relationship) for the Rpk parameter. The correlation between the contact angle and the parameters Rsk and Rpk is negative, in other words, an increase in the roughness parameters causes a decrease in the contact angle (increase in wettability). This is consistent with the Wenzel equation, which claims that an increase in surface roughness causes an increase in surface wettability (a decrease in the contact angle) for hydrophilic surfaces, for hydrophobic surfaces the relationship is inversely.

Considering distilled water as the measuring liquid, the greatest contact angle was measured for sample G produced during anodizing at a current density of 2 A/dm^2^, in an electrolyte at a temperature of 283 K, and was 95.33 ± 3.86°. The second sample with a contact angle exceeding 90° is sample A, the layer of which was formed during anodizing at a current density of 1 A/dm^2^ and an electrolyte temperature of 283 K, and the angle was 90.8 ± 2.7°. Both sample G and A are layers with hydrophobic properties. It follows from the above that low current density values combined with a short anodizing time (20 min) allow the production of a layer with high contact angles (low wettability).

**Figure 18:**
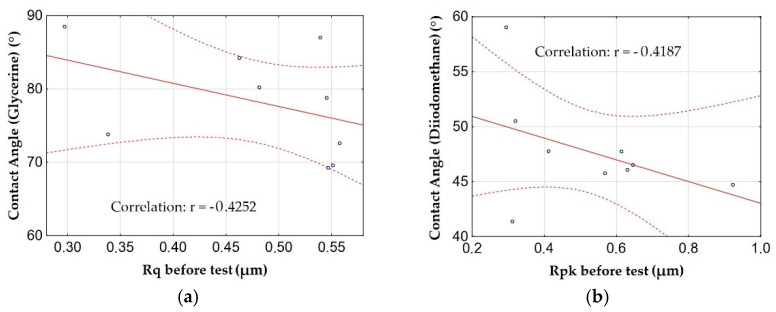
The relationship between the contact angle: (**a**) measured by glycerin and the Rq parameter before the test, (**b**) measured by diiodomethane and the Rpk parameter before the test.

Figure 18 shows the graphs of the relationship (correlation) between the contact angles measured with glycerin and diiodomethan, and the amplitude parameters and the load-bearing curve with the highest correlations.

The contact angle measured by glycerin and diiodomethane was found to be related to the amplitude parameters and the load capacity curve. There is a moderate correlation (significant relationship) between the contact angles for glycerin and diiodomethane and the Rq and Rpk parameters. The correlation is negative, which means an increase in contact angles with a decrease in Rq and Rpk parameters, similar to the measurements by water.

Table 8 shows the values of surface free energy (SFE) calculated using the Owens–Wendt method using the contact angles for distilled water and α-bromonaphthalene.

When analyzing the values of surface free energy (Table 8), the highest SFE value was observed for sample E anodized at a current density of 1 A/dm^2^ at an electrolyte temperature of 293 K (the highest value of mass loss of T7W material was demonstrated for this sample). The lowest SFE value was calculated for sample G produced at a current density of 2 A/dm^2^, at an electrolyte temperature of 283 K (the lowest coefficient of friction was demonstrated for this sample). Sample G has the highest contact angle for water and one of the highest for glycerin. The inverse proportionality of the surface free energy value to the value of contact angles was also noted.

**Figure 19:**
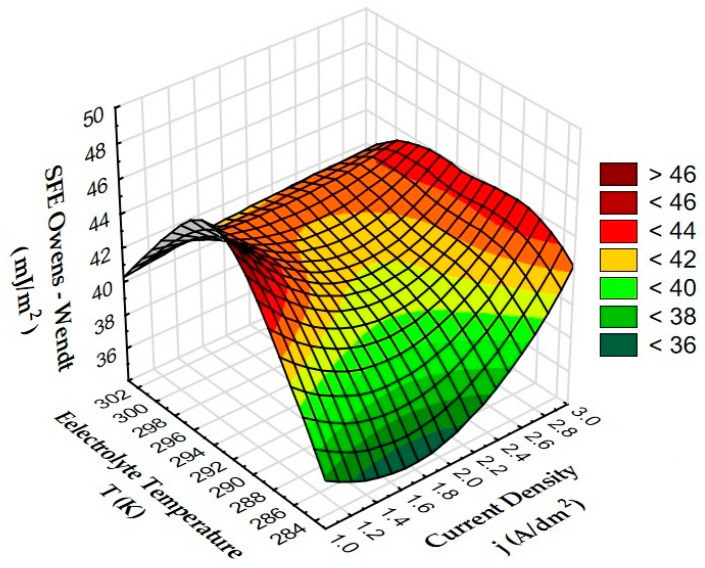
Dependence of SFE on electrolyte temperature and current density.

Figure 19 shows the dependence of the SFE on electrolyte temperature and current density.

Based on the analysis of the influence of the electrolyte temperature and current density on the surface free energy (Figure 11), it was found that high values (over 42 mJ/m^2^) were recorded for layers produced by anodizing at a current density of 3 A/dm^2^, in the full temperature range. The highest value was achieved for the lowest current values (1 A/dm^2^) and electrolyte temperature of 293 K. The lowest SFE values < 38 mJ/m^2^ occur at middle current density values and low electrolyte temperatures.

**Figure 20:**
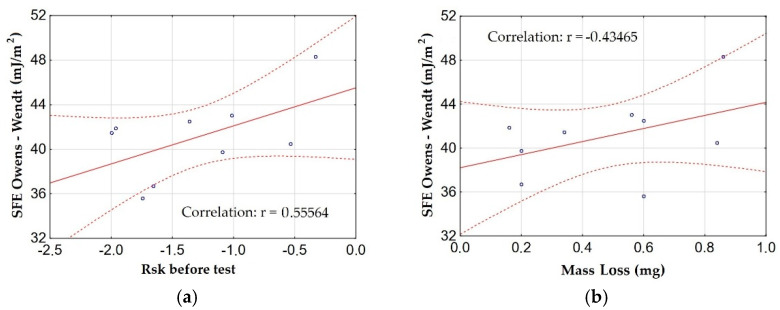
Relationship between SFE and: (**a**) Rsk parameter before the test, (**b**) mass loss of the material.

Figure 20 shows the graphs of the dependence (correlation) between the SFE calculated using the Owens–Wendt method and the Rsk parameter and the mass loss with the highest correlation.

When analyzing the correlation between the surface free energy, roughness parameters, and mass loss, a moderate correlation (significant relationship) with the Rsk parameter was found. The correlation is positive, which indicates the proportionality of the SFE value to the Rsk parameter, it is analogous to the values of the contact angles, the values of which are inversely proportional to the free surface energy. The increase in SFE also affects the mass loss of the material during friction. The analysis showed a moderate correlation, and thus a significant relationship between the surface free energy and material wear. The correlation is positive, which indicates the proportionality of the SFE value to the mass loss of the material.

## 4. Conclusions

- The research results and their analysis presented in the article confirm the legitimacy of anodizing the EN AW-5251 aluminum alloy in order to improve its sliding properties.
- The anodizing parameters used during the experiment significantly affect the thickness of the Al_2_O_3_ layers. Increasing the current density causes a significant increase in the thick-ness of the layer, as a result of increasing the electric charge, while the increase in temperature causes a decrease in the thickness of the layer, as a result of the increased secondary solubility of the Al_2_O_3_ layer by the electrolyte with a higher temperature.
- The correlation analysis also showed a significant relationship between the anodizing parameters and the surface roughness of the samples, which in turn affects the wear of the material and the resistance to motion. An increase in the Rsk parameter and a decrease in Rvk causes an increase in the consumption of T7W material, while a decrease in Rsk and an increase in Rvk contribute to a reduction in material consumption. An increase in the Rsk value also increases the friction coefficient. The differences in the roughness parameters result in a different character of the overlapping of the sliding film on the surface of the layers, which in turn translates into differences in material wear and movement resistance.
- The anodizing parameters and surface roughness also affect the wettability of the surface of the layers, which also affects the ability to form and maintain a sliding film. Low current density values (1 and 2 A/dm^2^) in combination with low electrolyte temperature (283 K) allow for the production of a layer with high contact angles (low wettability). The correlation between the contact angle and the Rsk and Rpk parameters is negative; in other words, an increase in the roughness parameters causes a decrease in the contact angle (increase in wettability).
- The analysis of friction and wear tests showed that the layer with hydrophobic properties, produced at a current density of 1 A/dm^2^ in an electrolyte at a temperature of 283 K, is the most favorable for sliding associations with T7W material.

## Figures and Tables

**Table 1:** Total experiment plan.

Sample	Controlled Factors			
	On a Natural Scale		On a Standard Scale	
	Current Density j (A/dm^2^)	Electrolyte Temperature T (K)	×1	×2
A	1	283	−1	−1
B	3	283	1	−1
C	1	303	−1	1
D	3	303	1	1
E	1	293	−1	0
F	3	293	1	0
G	2	283	0	−1
H	2	303	0	1
I	2	293	0	0

**Table 2:** List of Al_2_O_3_ layers thicknesses produced in anodizing process.

Sample	Oxide Layers Thickness d (μm)	Deviation (μm)
A	5.8	0.5
B	19.0	0.7
C	5.1	0.2
D	17.6	0.4
E	5.2	0.3
F	17.7	0.5
G	11.9	0.5
H	11.3	0.1
I	11.4	0.3

**Table 3:** The values of the coefficient of friction µ of the Al_2_O_3_ layers and of the aluminum alloy when working with the T7W material.

Sample	Coefficient of Friction µ
A	0.1425
B	0.1443
C	0.1661
D	0.1438
E	0.1463
F	0.1615
G	0.1419
H	0.1513
I	0.1564
Al	0.1932

**Table 4:** Mass loss of the T7W mandrel in association with the samples.

Sample	Mass Loss (mg)
A	0.20
B	0.60
C	0.20
D	0.34
E	0.86
F	0.56
G	0.60
H	0.16
I	0.84
Al	0.60

**Table 5:** Amplitude parameters and parameters of the load capacity curve of layers before and after friction.

Sample	Amplitude Parameters			Parameters of the Load Curve (Abott Fireston)		
	Rq (μm)	Rsk	Rku	Rk (μm)	Rpk (μm)	Rvk (μm)
Before test						
A	0.4625	−1.6561	8.1086	0.8090	0.2937	0.9324
B	0.5513	−1.3622	6.2085	1.0764	0.3112	0.9693
C	0.4815	−1.0896	12.1512	0.7486	0.9222	0.9337
D	0.5452	−1.9966	14.2198	0.8502	0.5683	1.2482
E	0.3381	−0.3301	11.8179	0.4928	0.6135	0.6071
F	0.5467	−1.0133	8.6509	0.9570	0.6454	0.9341
G	0.5392	−1.7443	5.4716	1.0826	0.3195	0.9328
H	0.5573	−1.9644	16.1986	0.7969	0.6299	1.1172
I	0.2970	−0.5330	7.3632	0.5634	0.4120	0.4280
Al	0.0765	−0.2227	9.8823	0.1415	0.0911	0.1162
After test						
A	0.4068	−1.6021	8.7518	0.6347	0.3303	0.9389
B	0.3700	−1.4232	6.9293	0.6591	0.2449	0.6995
C	0.4385	−2.3079	12.9483	0.5856	0.3199	0.8947
D	0.4178	−2.7333	14.1218	0.6194	0.2655	1.0170
E	0.1946	−0.4623	5.2914	0.3416	0.2375	0.2871
F	0.2332	−0.5208	4.7619	0.4419	0.2375	0.3654
G	0.4182	−1.9585	10.4411	0.7215	0.2762	0.7879
H	0.3809	−2.6221	14.4178	0.5178	0.2793	0.8692
I	0.1735	0.1649	4.2484	0.3442	0.2384	0.1948
Al	0.8276	−0.7317	6.2506	1.7494	0.9724	1.6857

**Table 6:** Contact angles of oxide layers and aluminum alloy measured by distilled water and α-bromonaphthalene.

Sample	Contact Angle (Distilled Water) (°)	Deviation (°)	Contact Angle (α-Bromonaphthalene) (°)	Deviation (°)
A	90.80	2.70	39.88	4.44
B	82.16	5.70	29.34	2.54
C	82.25	5.14	37.68	3.23
D	85.06	3.67	30.12	2.43
E	65.49	5.98	33.05	2.80
F	82.46	6.29	27.18	2.24
G	95.33	3.86	31.54	3.75
H	83.81	2.25	29.83	3.75
I	85.83	4.11	32.54	2.45
Al	71.59	4.14	36.12	2.36

**Table 7:** Contact angles of oxide layers and aluminum alloy measured by glycerin and diiodomethane.

Sample	Contact Angle (Glycerin) (°)	Deviation (°)	Contact Angle (Diiodomethane) (°)	Deviation (°)
A	84.25	7.69	59.05	2.26
B	69.59	3.01	41.38	3.19
C	80.22	5.24	44.74	6.38
D	78.80	1.36	45.76	7.09
E	73.80	2.55	47.74	7.75
F	69.27	2.89	46.51	3.65
G	87.03	4.10	50.52	4.98
H	72.60	4.04	46.09	5.93
I	88.54	2.76	47.77	3.02
Al	80.67	3.67	52.19	2.14

**Table 8:** Free surface energy of Al_2_O_3_ layers for glycerin and diiodomethane.

Sample	SFE Owens–Wendt (mJ/m^2^)
A	36.68
B	42.49
C	39.75
D	41.46
E	48.31
F	43.04
G	35.61
H	41.87
I	40.48
Al	49.81

## Data Availability

The data presented in this study are available on request from the corresponding author. The data are not publicly available due to insufficient space to insert data.
